# Malaria and Fetal Growth Alterations in the 3^rd^ Trimester of Pregnancy: A Longitudinal Ultrasound Study

**DOI:** 10.1371/journal.pone.0053794

**Published:** 2013-01-11

**Authors:** Christentze Schmiegelow, Daniel Minja, Mayke Oesterholt, Caroline Pehrson, Hannah Elena Suhrs, Stéphanie Boström, Martha Lemnge, Pamela Magistrado, Vibeke Rasch, Birgitte Bruun Nielsen, John Lusingu, Thor G. Theander

**Affiliations:** 1 Centre for Medical Parasitology, Institute of International Health, Immunology, and Microbiology, University of Copenhagen, Copenhagen, Denmark; 2 National Institute for Medical Research, Tanga Medical Research Center, Tanga, Tanzania; 3 Department of Medical Microbiology, Radboud University Nijmegen Medical Centre, Nijmegen, The Netherlands; 4 Department of Public Health, University of Copenhagen, Copenhagen, Denmark; 5 Department of Immunology, Wenner-Gren Institute, Stockholm University, Stockholm, Sweden; 6 Department of Obstetrics and Gynecology, Odense University Hospital, Odense, Denmark; 7 Department of Obstetrics and Gynecology, Aarhus University Hospital, Skejby, Denmark; 8 Department of Infectious Diseases, Copenhagen University Hospital, Copenhagen, Denmark; Mahidol University, Thailand

## Abstract

**Background:**

Pregnancy associated malaria is associated with decreased birth weight, but in-utero evaluation of fetal growth alterations is rarely performed. The objective of this study was to investigate malaria induced changes in fetal growth during the 3^rd^ trimester using trans-abdominal ultrasound.

**Methods:**

An observational study of 876 pregnant women (398 primi- and secundigravidae and 478 multigravidae) was conducted in Tanzania. Fetal growth was monitored with ultrasound and screening for malaria was performed regularly. Birth weight and fetal weight were converted to z-scores, and fetal growth evaluated as fetal weight gain from the 26th week of pregnancy.

**Results:**

Malaria infection only affected birth weight and fetal growth among primi- and secundigravid women. Forty-eight of the 398 primi- and secundigravid women had malaria during pregnancy causing a reduction in the newborns z-score of −0.50 (95% CI: −0.86, −0.13, *P* = 0.008, multiple linear regression). Fifty-eight percent (28/48) of the primi- and secundigravidae had malaria in the first half of pregnancy, but an effect on fetal growth was observed in the 3^rd^ trimester with an OR of 4.89 for the fetal growth rate belonging to the lowest 25% in the population (95%CI: 2.03–11.79, *P*<0.001, multiple logistic regression). At an individual level, among the primi- and secundigravidae, 27% experienced alterations of fetal growth immediately after exposure but only for a short interval, 27% only late in pregnancy, 16.2% persistently from exposure until the end of pregnancy, and 29.7% had no alterations of fetal growth.

**Conclusions:**

The effect of malaria infections was observed during the 3^rd^ trimester, despite infections occurring much earlier in pregnancy, and different mechanisms might operate leading to different patterns of growth alterations. This study highlights the need for protection against malaria throughout pregnancy and the recognition that observed changes in fetal growth might be a consequence of an infection much earlier in pregnancy.

## Introduction

Pregnancy associated malaria (PAM) caused by *Plasmodium falciparum* is associated with maternal anemia [1], stillbirth [2], preterm delivery [3], [4] and decreased birth weight (BW) [3]–[7]. Furthermore, PAM might have long-term health consequences for the newborn due to an increased risk of non-communicable diseases among adults born with low birth weight (BW<2500 g) (LBW) and poor intrauterine growth [8], [9]. Maternal immunity to PAM is acquired after malaria infections over consecutive pregnancies leaving women in their first pregnancies most at risk [1], [10], [11]. *P. falciparum* invades the erythrocytes, and the effect on BW has been attributed to maternal anemia [5], [12] and the sequestration of infected erythrocytes in the placenta [13]–[17]. It is still unknown, when during pregnancy the woman and fetus are most vulnerable to PAM [7], [18]–[23]. However, many studies indicate that the first half of pregnancy is a particularly vulnerable period [7], [18]–[20], [22], [23], but little is known of when and how fetal growth is affected. LBW is used as a measure of the consequence of PAM [4], [18], [19], [21], but this is misleading as an indicator of altered fetal growth since LBW encompasses both newborns having suffered poor intrauterine growth as well as preterm newborns without growth impairment [24]. A better way to evaluate fetal growth is therefore to report individual changes over time expressed as z-score changes (deviation of weight from the gestational age (GA) adjusted mean) [25], [26] or fetal weight gain [27], [28].

To ensure that preventive strategies are targeting the most vulnerable time-points in pregnancy a better understanding of fetal growth alterations after PAM is warranted [10]. The objective of this study was to investigate the effect of PAM on fetal growth in the late half of pregnancy by using trans-abdominal ultrasound among a cohort of pregnant women in northeastern Tanzania. We here present evidence of fetal growth alteration in the 3^rd^ trimester of pregnancy after a malaria infection.

## Methods

Ethical approval was granted by the Tanzania Medical Research Coordinating Committee (MRCC) (18^th^ of April 2008, reference number NIMR7HQ/R.8a/Vol. IX/688). All procedures were conducted in accordance with the Declaration of Helsinki and Good Clinical and Laboratory Practices. All participants gave informed written consent.

The study was conducted in Korogwe District, northeastern Tanzania. Malaria transmission is holoendemic, but has lately shown a remarkable decline [29]. Pregnant women attending the Reproductive and Child Health clinic of Korogwe District Hospital (KDH) or the Lwengera, Kerenge and Ngombezi Dispensaries were enrolled from September 2008 until March 2010 and followed throughout pregnancy. The last delivery occurred in October 2010. The study design has been reported previously [30], [31]. In short, inclusion criteria were: GA≤24 weeks determined by ultrasound, living in an accessible area of Korogwe District for >6 months, and willing to give birth at KDH. Women with preeclampsia and/or twins in the current pregnancy, and fetuses/newborns with severe malformations were excluded from the analysis, since these conditions can severely affect fetal growth [32], [33]. In analyses including BW, stillbirths were omitted. Sensitization campaigns were performed in the villages to reduce selection bias. After inclusion, women were booked for three antenatal visits (ANV) at a GA of 26 (ANV2), 30 (ANV3), and 36 (ANV4) weeks. If needed due to illness women attended extra clinic visits. Maternal age, anthropometric measures, obstetric history, educational level (≤primary school; ≥secondary school), HIV status, and hemoglobin level (Sysmex hematological analyzer®, Kobe, Japan) were documented. The project team performed all investigations except screening for maternal HIV infection which governmental nurses performed only three days a week. Women not completing follow-up were therefore less likely to have their HIV status determined. Transport was offered at the time of delivery and 77.2%, 5.3%, and 17.6% delivered at KDH, another health facility, and at home, respectively. Home visits were performed within one week of the bookings or the estimated date of delivery, if women failed to report at KDH.

At all visits venous blood was collected and at delivery both venous and placental blood. Malaria was diagnosed using rapid diagnostic test (RDT) (Parascreen™ Zephyr Biomedicals, Goa, India, Paracheck Pf® Orchid Biomedical Systems, Goa, India or ParaHIT® Span diagnostics Ltd, Surat, India) [34]. Thick and thin blood smears were prepared and evaluated at the end of the study. Women were therefore treated based on the RDT results with Artemether-Lumefantrine (Coartem® Dispersible, Norvatis Corporation Suffern, New York, USA) or Quinine (Quinine sulfate coated tablets, ELYS chemical Industries Ltd, Nairobi, Kenya) (infections occurring in 1^st^ trimester). Women with symptoms or sign of malaria had an immediate blood smear investigation in addition to the RDT. Infections requiring hospital admission were treated with Quinine (Quinine Dihydrochloride Injection BP, Healthcare PVT Ltd, Mumbai India). RDT positive women, who had been RDT positive within two weeks before the ANV, also had an immediate blood smear examination. Parasite HRP-2 antigen can circulate in the blood stream after clearance of the malaria parasites, and only women who were consistently blood smear positive were treated [35]. Blood smears were stained with Giemsa, and asexual parasites counted against 200 (500 if parasite count was <10) leucocytes. One hundred thick film fields were read before a slide was declared negative. The malaria infection was considered symptomatic if the axillary temperature was above 37.5C.

Intermittent preventive treatment for malaria (IPTp) with sulfadoxine-pyrimethamine (1500 mg/75 mg) (SULPHADAR®, Shelys Pharmaceutical Ltd., Dar es Salaam, Tanzania) was given as directly observed treatment; 1^st^ dose in the 2^nd^ trimester after a GA of 20 weeks and 2^nd^ dose in the 3^rd^ trimester, at least four weeks apart. If included early in pregnancy the 1^st^ dose was given at an extra clinic visit at a GA of 20. If having received a 1^st^ dose of IPTp before inclusion, but earlier than recommended by WHO (2^nd^ trimester when quickening is felt [36]), the woman received a 2^nd^ dose at week 20 and a 3^rd^ dose in the 3^rd^ trimester. According to the national program, voucher for procuring a bednet was provided and bednet use inquired.

Trans-abdominal ultrasound examination was performed at inclusion, ANV2, ANV3, and ANV4 (Sonosite TITAN®, US High resolution ultrasound system, 5–2 MHz C60 abdominal probe, Bothell, Washington state, USA). At inclusion GA was estimated using crown-rump length (crown-rump length<75 mm) [37]) or head circumference of the fetus [38]. If the GA was <11 weeks a new GA estimation was performed at 11–16 weeks of gestation. At ANV2, 3, and 4 fetal weight (EFW) were estimated using the Hadlock algorithms based on head circumference, abdominal circumference and femur length [39]. The performance of the Hadlock algorithms in this population has been reported previously [31]. All investigations were performed by the author CS, assisted by a trained Tanzanian midwife.

To account for dependency of growth velocity on initial weight, fetal growth was evaluated as the relative weight gain in g/week*kg in four fixed growth intervals of at least 14 days [28], [40] representing different time-points in pregnancy (ANV2-ANV3 (late 2^nd^ trimester/); ANV3-ANV4 (early 3^rd^ trimester); ANV4-Delivery (late 3^rd^ trimester); ANV3-Delivery (entire 3^rd^ trimester)) using the fetal weight (kg) in the beginning of each growth interval as the reference [27], [28], [41]. Relative weight gain was furthermore dichotomized as the lowest 25% or the highest 75%. At delivery BW, head and abdominal circumference, sex, and placental weight were documented. BW measured within 24 hours of delivery using a spring scale (Fazzini®, accuracy = 50 g, Vimodrone, Italy) (KDH, until July 2009 and all examinations at home) or a digital strain gauge scale (ADE®, accuracy = 10 g, Hamburg, Germany) (KDH, after July 2009) were considered eligible.

To exclude variability due to GA and sex, EFW and BW were converted to z-scores using sex-specific standard weight charts developed from healthy pregnancies in the same cohort as a reference [31].

Changes in z-score over time were investigated to further evaluate alterations in fetal growth. Only offspring with an eligible BW and data from at least two growth intervals were evaluated. Primarily, the growth intervals ANV2-ANV3, ANV3-ANV4, ANV4-Delivery were used for evaluation. Alternative growth intervals (ANV2-ANV4, ANV3-Delivery) were used if a woman had incomplete attendance. Changes in z-scores (Δz) were calculated for the malaria negative group and used as a reference. Theoretically, a normally growing fetus will have a Δz close to zero, whereas a Δz>0 represents excess fetal weight gain and a Δz<0 insufficient weight gain. Evaluation of changes in z-scores can be affected by the fetal pulsatile growth pattern [42] and to limit false-positives the 25^th^ centile for Δz was used as a cut-off to define insufficient weight gain. The effect of malaria was categorized in four groups: 1) Normal growth as Δz ≥25^th^ centile in all growth intervals following a malaria infection, 2) Immediate effect as Δz <25^th^ centile in the growth interval when the infection occurred or in the first growth interval after a malaria infection, followed by growth intervals where Δz ≥25^th^ centile, 3) Late effect as a Δz<25^th^ centile in a growth interval not immediately following the infection, and 4) Persistent effect as a Δz <25^th^ centile immediately after a malaria infection and until the end of pregnancy.

For analysis, women were considered to be malaria negative if they never had malaria based on RDT and microscopy results from the time they were enrolled. Women, who contracted malaria (RDT and/or microscopy positive), were considered to belong to the malaria group from when they were diagnosed until delivery. In growth intervals preceding the time of detection of the malaria infection they were excluded from the analysis. Hence, women contracting malaria between ANV2 and ANV3 were not included in analyses of the growth interval ANV2-ANV3, but considered malaria positive in all other growth intervals.

### Statistics

All data were documented and validated using Microsoft Office Access 2003. Statistical analyses were performed in Stata 10 (Stata Corporation) using, when appropriate, Chi^2^ test, Fisher’s exact test, Mann Whitney ranksum, and Student t-test, and all with two-sided *P*-values. The effect of malaria on BW (as z-score) was investigated using multiple linear regression and on relative weight gain (dichotomized as lowest 25% and highest 75%) using multiple logistic regression. Crude and adjusted coefficients/odds ratios were calculated. Factors with a *P*<0.20 in univariate analysis were entered into the multivariate models. Using a step-wise backward elimination approach final models were obtained including variables with a *P*<0.10. A *P<*0.05 was considered significant. Final models only included women without missing values.

## Results

In total, 1171 pregnant women were screened and 995 women met the inclusion criteria. Of these 21 women had miscarriage, 11 withdrew consent, 34 were lost to follow-up, 5 moved out of the district, and 924 women completed follow-up. Of these, 48 were excluded due to (some with multiple conditions) preeclampsia (28), twin pregnancy (16), and/or severe congenital malformation (6), leaving 876 women-newborn pairs for analysis. The included women were more often multigravidae, had a known HIV status and lower educational level compared to the excluded women (Supplementary Table 1). In total, 18 stillbirths occurred.

**Table 1:** Characteristics of the 876 mother-newborn pairs eligible for analysis stratified by gravidity.

		Primi- and secundigravidae (n = 398)		Multigravidae (n = 478)	
		Total	Median (range)/n (%)	Total	Median (range)/n (%)
GA at inclusion (days)		398	128 (42–168)	478	131 (50–168)
GA at inclusion <14 weeks		398	76 (19.1)	478	77 (16.1)
GA at 14–24 weeks		398	322 (80.9)	478	401 (83.9)
Age (y)		398	22 (14–37)	477	30 (19–47)
Education ≤primary level		396	305 (77.2)	474	451 (95.2)
Ethnicity	Sambaa	398	183 (46.0)	477	244 (51.2)
	Zigua		59 (14.8)		104 (21.8)
	Pare		31 (7.8)		21 (4.4)
	Bondei		18 (4.5)		17 (3.6)
	Other^a^		107 (26.9)		91 (19.1)
Maternal height (cm)		395	158 (143–183)	476	157 (142–187)
Maternal weight at incl. (kg)		395	53 (37–126)	475	54 (37–100)
MUAC <23cm at inclusion^b^		397	49 (12.3)	477	31 (6.5)
Malaria during pregnancy^c^		398	48 (12.1)	478	24 (5.0)
Malaria before ANV2^d^		48	28 (58.3)	24	7 (29.2)
Median parasitaemia (IE/ul)^d^		32	2090 (89–390.749)	8	4163 (39–45.760)
Single malaria infection^d^		48	39 (81.2)^e^	24	22 (91.7)^f^
Received IPTp≥2 times		398	383 (96.2)	478	463 (96.9)
Never used bednet		398	32 (8.0)	478	13 (2.7)
Maternal HIV infection		368	9 (2.5)	439	32 (7.3)
Male newborn		393	194 (49.4)	470	239 (50.9)
Placental weight (g)^g^		310	587±142	346	598±150

a Other include various ethnic groups representing <2% of the women (not stratified by gravidity).

b MUAC<23 cm was used as a marker for poor nutritional status [53].

c Comparison of malaria prevalence using Chi^2^ test p<0.001.

d Only includes malaria positive women.

e 17% (8/48) had two infections and 2% (1/48) three infection.

f 8% (2/24) had two infections.

g Mean±SD.

Abbreviations: ANV = antenatal visit,, GA = gestational age, G = gram, HIV = human immunodeficiency virus, IE = infected erythrocytes, Incl. = inclusion, IPTp = intermittent preventive treatment in pregnancy, MUAC = mid upper arm circumference, N = number, ul = microliter, Y = year.

Characteristics of the 876 mother-newborn pairs are shown in Table 1 and 2. In total, 398 primi- and secundigravidae and 478 multigravidae completed follow-up. Malaria during pregnancy was diagnosed in 8.2% (72/876) of the women, and was more common among primi- and secundigravidae than among multigravidae (*P*<0.001, Chi^ 2^ test) (Table 1). Except for rarer use of bednets (primi- and secundigravidae) and IPTp (multigravidae), higher risk of anemia (primi- and secundigravidae) and lower placental weight (primi- and secundigravidae) there were no difference in baseline characteristics between the malaria positive and negative groups (Supplementary Table S2 and S3). None of the multigravidae, and six of the primi- and secundigravidae had a symptomatic malaria infection. Hereof, three women were admitted to the hospital for treatment. In total, 40 women were slide positive at some point and 32 women were only RDT positive but never slide positive. Among the women who were slide positive, five primi- and secundigravidae were RDT negative, and hence not treated for their malaria infection, because microscopy were performed retrospectively. None of these women were RDT positive at any of the consecutive visits. Six women (four primigravidae and two multigravidae) were RDT positive, but microscopy negative, within 14 days of treatment for their initial malaria infection and were not treated again. Details on the malaria infections are given in Table 1 and characteristics of all malaria positive women are available as Supplementary Table S4.

**Table 2:** Fetal weight, fetal growth, birth weight and gestational age at delivery.

		Primi- and secundigravidae (n = 398)		Multigravidae (n = 478)	
		n	Median (range)/Mean±SD	n	Median (range)/Mean±SD
Fetal weight (g)	ANV2	382	884 (605–1246)	461	883 (648–1253)
	ANV3	376	1469 (1035–1942)	461	1481 (1124–2167)
	ANV4	352	2575 (1894–3345)	440	2616 (1593–3340)
Fetal growth (g/week)^a^	ANV2-3	365	146±23	443	151±23
	ANV3-4	336	187±29	427	190±30
	ANV4-Del	282	148±83	357	154±91
Birth weight (g)^b^	Malaria pos.	**37**	**2910 (2000–4000)**	16	3415 (3000–3500)
	Malaria neg.	**298**	**3100 (1200–4500)**	387	3180 (900–4510)
Z-score at Del.a, c	Malaria pos.	**36**	**−0.63±1.13**	16	0.34±1.01
	Malaria neg.	**298**	**−0.06±1.08**	383	0.02±1.09
GA at Del. (days)^d^	Malaria pos.	47	278 (246–302)	24	280 (267–299)
	Malaria neg.	348	280 (209–299)	453	280 (178–308)

a Mean±SD (parametric distribution).

b Mann-Whitney, comparison of malaria negative and positive, p = 0.005 for primi- and secundigravidae, p = 0.32 for multigravidae.

c Students t-test, comparison of malaria negative and positive, p = 0.003 for primi- and secundigravidae, p = 0.26 for multigravidae.

d Mann-Whitney, comparison of malaria negative and positive, no significant difference.

Abbreviations: ANV = antenatal visit, Del. = delivery, GA = gestational age, G = gram, N = number, Neg. = negative, Pos. = positive.

The majority of ANV2, 3 and 4 was conducted at a GA of 26 (698/843 (82.3%) estimated fetal weights (EFW)), 30 (727/837 (86.9%) EFW) and 36 (688/792 (86.9%) EFW) weeks, respectively. Women showing early or late for their bookings had fetal weight estimated within 1.5 weeks of these GA. The timing of the ANV was comparable for the malaria positive and malaria negative women (Supplementary Table S2 and S3).

Among primi- and secundigravidae malaria infection resulted in a decrease in median BW (2910 g (malaria positive) vs. 3100 g (malaria negative), *P* = 0.005, Mann-Whitney), whereas malaria infection did not affect the BW among multigravidae. Furthermore, the newborns Z-score on BW was statistically significant lower among the malaria positive compared to the malaria negative primi- and secundigravidae (−0.63 (malaria positive) vs. −0.06 (malaria negative), *P* = 0.003, Students t-test). Malaria infection did not affect the GA at delivery (Table 2).

Alterations in fetal growth patterns following malaria infections were further investigated among primi-and secundigravidae by evaluating the relative fetal weight gain in the four growth intervals in the late half of pregnancy (ANV2-ANV3, ANV3-ANV4, ANV4-Delivery, ANV3-Delivery) (Table 3). In univariate analyses relative fetal weight gain was statistical significantly reduced in the 3^rd^ trimester of pregnancy in the malaria positive group compared to the malaria negative group. The relative weight gain had unequal variance and we therefore dichotomized the women into those with a relative fetal weight gain belonging to the lowest 25% and those with a weight gain belonging to the highest 75%, to allow adjusting for confounding factors using multiple logistic regression. In the 3^rd^ trimester, in the univariate analyses, there were a statistical significantly higher proportion of fetuses belonging to the lowest 25% among the malaria positive compared to the malaria negative group (Table 4). Fetal growth is affected by maternal anthropometry and fetal sex [42]–[44]. We therefore investigated the effect of malaria on fetal growth and BW after adjusting for possible confounding factors (Table 5). The characteristics listed in Table 1, GA at the ANVs and at delivery and place of delivery were evaluated as possible confounding factors (Supplementary Table S5 and S6). After adjustment, Z-score at birth was still affected by malaria with a decrease in z-score of −0.50 after exposure (95% CI: −0.86, −0.13; *P* = 0.008, multiple linear regression). Malaria infection was also associated with a relative fetal weight gain belonging to the lowest 25% in all three growth intervals in the 3^rd^ trimester after adjusting for confounding factors (ANV3-ANV4 adj. odds ratio (OR): 2.72, 95% CI: 1.18–6.23, *P* = 0.019; ANV4-Delivery adj. OR: 2.48, 95% CI: 1.04–5.91, *P* = 0.040; ANV3-Delivery adj. OR: 4.89, 95% CI: 2.03–11.79, *P*<0.001) (Table 5). Only considering women with a single infection, adjusting for number of infections, or excluding women who did not receive malaria treatment did not alter the results (data not shown).

**Table 3:** The effect of malaria during pregnancy on fetal growth, given as relative fetal growth (g/week*kg), among primi- and secundigravidae.

		Malaria positive^a^ (n = 48)		Malaria negative^a^ (n = 350)		
		Total	Median (95% CI)	Total	Median (95% CI)	*P* b
EFW at ANV2^c^		46	884 (861–903)	336	884 (874–897)	0.80
As g/week*kg	ANV2-3	29	159 (151–177)	321	165 (161–170)	0.91
	ANV3-4	**32**	**121 (113–129)**	**296**	**130 (128–133)**	**0.046**
	ANV4-Del	**27**	**40 (29–54)**	**253**	**57 (53–62)**	**0.017**
	ANV3-Del	**30**	**94 (87–128)**	**281**	**119 (116–125)**	**0.006**

a Women were considered to be malaria negative if they never had malaria based on RDT and microscopy results from the time they were enrolled. Women, who contracted malaria, were considered to belong to the malaria group from when they were diagnosed until delivery. When investigating a particular growth interval, women who only contracted malaria after the particular growth interval were excluded from that specific analysis.

b Mann Whitney test.

c The malaria positive group include all women contracting malaria during pregnancy.

Abbreviations: ANV = antenatal visit, CI = confidence interval, Del = delivery N = number.

**Table 4:** Proportion of primi and secundigravidae with a fetal weight gain belonging to the lowest 25%, stratified by malaria positivity.

		Malaria positive^a^ (n = 48)		Malaria negative^a^ (n = 350)		
		Total	% (95% CI)	Total	% (95% CI)	*P* b
Lowest 25%	ANV2-3	29	26 (13–47)	321	24 (19–29)	0.67
	ANV3-4	**32**	**44 (26–62)**	**296**	**23 (18–28)**	**0.009**
	ANV4-Del	**27**	**41 (22–61)**	**253**	**23 (18–29)**	**0.047**
	ANV3-Del	**30**	**53 (34–72)**	**281**	**21 (17–27)**	**<0.001**

a Women were considered to be malaria negative if they never had malaria based on RDT and microscopy results from the time they were enrolled. Women, who contracted malaria, were considered to belong to the malaria group from when they were diagnosed until delivery. When investigating a particular growth interval, women who only contracted malaria after the particular growth interval were excluded from that specific analysis.

b Chi^2^ test.

Abbreviations: ANV = antenatal visit, CI = confidence interval, Del = delivery N = number.

**Table 5:** The effect of malaria among primi- and secundigravidae on z-score of birth weight, relative weight gain from ANV3 to ANV4, ANV4 to delivery, and ANV3 to delivery.

		n	Coeff./OR	95% CI	*P*
Change in Z-score at delivery^a^		**285**	**−0.50**	**−0.86, −.13**	**0.008**
Relative weight gain belonging to lowest 25% (OR)	ANV3-A4^b^	**327**	**2.72**	**1.18, 6.23**	**0.019**
	ANV4- Del^c^	**258**	**2.48**	**1.04, 5.91**	**0.040**
	ANV3- Del^d^	**282**	**4.89**	**2.03, 11.79**	**<0.001**

Coefficient and ORs adjusted for confounding factors. Relative weight gain was dichotomized as belonging to the lowest 25% or highest 75% of the distribution observed among primi- and secundigravidae.

a Multiple linear regression, adjusted for maternal weight at inclusion, maternal weight gain from inclusion-ANV3 (median gain 198 g/week, 95% CI: 170–230 g/week), maternal weight gain from ANV3-ANV4 (median gain 250 g/week, 95% CI: 168–326 g/week), placental weight, and place of delivery, 32 malaria positive and 253 malaria negative.

b Multiple logistic regression, adjusted for MUAC at inclusion and GA at ANV3, 32 malaria positive and 295 malaria negative.

c Multiple logistic regression, adjusted for placental weight, 25 malaria positive and 233 malaria negative women d) Multiple logistic regression, adjusted for GA at ANV3, GA at delivery, sex of newborn and placental weight, 28 malaria positive and 254 malaria negative.

Abbreviations: ANV = antenatal visit, CI = confidence interval, Coeff. = coefficient, Del = delivery N = number, OR = odds ratio.

**Figure 1:**
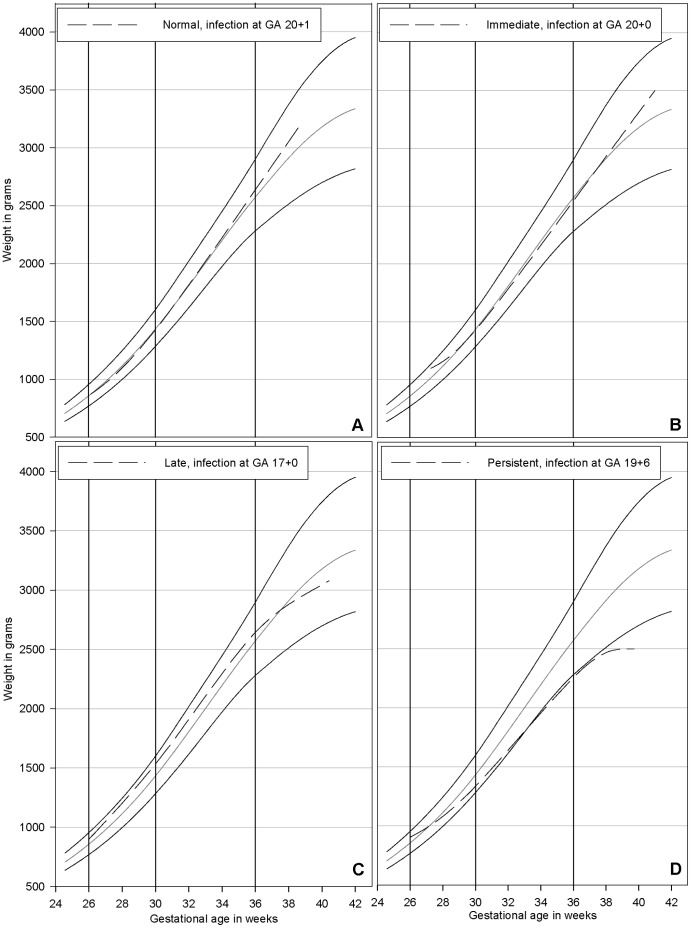
Different growth patterns after a malaria infection. The four individual growth patterns are superimposed on a weight chart developed from healthy pregnancies in the cohort [31]. Normal growth observed after an infection at gestational age (GA) 20+1 (panel A). Immediate effect observed after an infection at GA 20+1 with an initial decline in growth observed in the growth interval antenatal visit (ANV)2– ANV3 followed by persistently normal growth until delivery (panel B). Late effect observed after an infection at a GA 17+0 with normal growth until ANV4 and thereafter a decline in growth until delivery (panel C). Persistent effect observed after an infection at GA 19+6 with decline in growth throughout pregnancy (panel D). The solid vertical lines indicate the timing of the three ANV. In panel B the ANV2 occurred slightly delayed at a GA of 27+2. The solid black and grey lines represent the 90^th^, 50^th^, and 10^th^ percentile.

To further elucidate how and when fetal growth in the last half of pregnancy was affected by malaria we evaluated the change in z-score over time. To illustrate the importance of gravidity when assessing the consequences of malaria on fetal growth multigravidae were also included in these analyses. Fifty-two of the 72 women (72.2%) with malaria had an eligible BW and data available for at least two growth intervals. Among the primi- and secundigravid women, the majority (26/37) experienced alterations in fetal growth after a malaria infection. An equal distribution between an immediate, a late and a persistent effect on fetal growth was observed. Among the majority of the multigravid women (10/15) the fetal growth was not affected. Furthermore, a persistent and a late alteration of fetal growth seemed to result in the lowest BW (Table 6). In Figure 1 examples of different growth patterns are illustrated.

**Table 6:** Patterns of fetal growth following a malaria infection evaluated using Δz for the individual offspring.

			Primi- and secundi	Multigravidae	All
Type of growth		Total^a^	n (%)	n (%)	Median BW (g) (95% CI)
Normal^b^		21	11/37 (29.7)	10/15 (66.6)	3300 (2994–3500)
Abnormal^c^	Immediate	12	10/37 (27.0)	2/15 (13.3)	2940 (2610–3490)
	Late^d^	11	10/37 (27.0)	1/15 (6.7)	2850 (2579–3023)
	Persistent	8	6/37 (16.2)	2/15 (13.3)	2705 (2338–3163)
Total		52	37	15	

a Only fetuses with data from at least two growth intervals and an eligible birth weight were included. Fifty-two of the 72 malaria exposed met these criteria. Forty had all three growth intervals available, one only ANV2-ANV4 and ANV4-Del, eight ANV2-ANV3 and ANV3-Del, and three ANV3-ANV4 and ANV4-Del.

b Normal growth = Δz ≥25^th^ centile in all growth intervals following a malaria infection.

c Immediate effect = Δz <25^th^ centile in the growth interval when the infection occurred or in the first growth interval after a malaria infection, followed by growth intervals where Δz ≥25^th^ centile; Late effect = Δz<25^th^ centile in a growth interval not immediately following the infection; Persistent effect = Δz <25^th^ centile immediately after a malaria infection and until the end of pregnancy.

d The effect was observed min. 6 weeks after the infection.

Abbreviation: BW = birth weight, CI = confidence interval, G = gram, N = number.

Among primi- and secundigravidae, there was a trend towards decreased head circumference at birth (Supplementary Table S2), decreased fetal abdominal growth from ANV3-Delivery, and decreased growth of the fetal head from ANV2-ANV3 and ANV3-ANV4 in the malaria positive compared to the malaria negative group (data not shown).

## Discussion

This study provides one of the first comprehensive analyses of fetal growth alterations associated with PAM by using ultrasound.

Malaria infections affected fetal growth among primi- and secundigravidae. Many studies report that primigravid women experience the most severe consequences of PAM [5], [45]. Malaria transmission has dramatically declined over the last years in the study area [29] and this might explain why also secundigravidae were of increased risk. More importantly, fetal growth was still altered after malaria infection despite the fact that RDT positive women were treated for malaria and more than 96% of all the women received at least two doses of IPTp. Women were considered as having been exposed to malaria if they had a positive RDT even though they were negative by microscopy. These samples have subsequently been analyzed by PCR [35]. Among primi- and secundigravidae eight were positive by RDT and negative by PCR (Supplementary Table S4). Excluding these women from the analyses or considering them as malaria negative did not alter the results (data not shown).

The relative fetal weight gain was significantly decreased among malaria positive primi- and secundigravidae compared to uncomplicated pregnancies. The effect of malaria was observed regardless of whether early, late, or the entire 3^rd^ trimester was evaluated. Furthermore, the newborns’ z-score was significantly decreased among the malaria exposed compared to the unexposed, indicating that a disproportional change in growth velocity occurred in the 3^rd^ trimester leading to the PAM induced decrease in BW previously reported [3]–[7]. Although, the decrease only was statistically significant in the 3^rd^ trimester, the data (Table 3) indicates that fetal growth was also decreased in late 2^nd^ trimester and that there might be trend of increasing difference in fetal growth over time, but this could not be confirmed statistically. Rijken *et al* [46] showed that malaria can cause growth restriction in the 2^nd^ trimester. This could cause an underestimation of the GA in the malaria positive group resulting in a less apparent effect of malaria on growth. This as well as the size of the study might have resulted in insufficient statistical power to detect a difference in late 2^nd^ trimester. Malaria was not associated with perinatal mortality [30], and stillborns were excluded from analyses were BW were included. Abnormal fetal growth resulting in stillbirth should therefore not have affected the results.

Upon investigating the individual alterations in fetal growth after malaria exposure, four distinct patterns were identified. Again, the most pronounced effect was observed among primi- and secundigravidae. Our results indicated that late and persistent alterations resulted in the lowest BW. The sample size was limited, and these results will need to be confirmed in future studies.

Most infections in our cohort were detected at inclusion (1^st^ and 2^nd^ trimester). Previous findings indicate that the timing of the infection matters [7], [18]–[22], with early infections being more harmful than late [7], [18]–[20], [22], [23]. In the 1^st^ and 2^nd^ trimester the placental development is particularly sensitive to pathology, and if the trophoblast invasion of the maternal spiral arteries are disrupted, current and later placental function can be impaired [47]. PAM has been speculated to disrupt trophoblast invasion [16], [17] and been associated with decreased placenta volume [48] and changes in utero-placental and umbilical blood flow [13], [49]. Sequestration of infected erythrocytes can also cause impaired placental function due to an inflammatory response and structural [15] as well as functional changes of the syncytiotrophoblast layer [14], [16]. A recent study by Griffin *et al* [49] reported changes in umbilical blood flow in the 3^rd^ trimester after infections occurring before a GA of 20 weeks. This indicates that the effect of PAM might be observed with considerable delay, which is in line with the pattern observed in our study. In the 3^rd^ trimester when nutrient requirement of the fetus is at its highest level, insufficient placental development and function could have severe consequences [16], [47]. Histopathological data of the placenta was not available in this study, but our data indicate that different mechanisms operate in different individuals leading to none, immediate, late or persistent alteration of fetal growth. Further investigations of the women’s immunological profiles and studies combining in-utero monitoring of fetal growth with histopathological investigations might clarify why different growth alterations arise. Partial immunity could explain some of the variation in the growth alterations after malaria infections.

Among the primi- and secundigravidae the majority of the infections were detected at the time of inclusion or just hereafter, and before the first dose of IPTp (Supplementary Table S4). A recent study provided evidence that IPTp administered early during pregnancy is more protective of LBW than late administration [50]. This and the current study highlights the importance of prescribing IPTp as early as possible, and the importance of identifying interventions that can diminish the risk of infection before the first antenatal visit. Alternatively, efforts should concentrate on promoting early antenatal attendance employing the current preventive measures with a “diagnose and treat” approach.

Infection with malaria prior to inclusion in the study might affect fetal growth, but reliable data on this was not available. However, including GA at inclusion in the multiple regression models did not alter the results (data not shown). In future studies, inclusion as soon as possible after conceiving should be sought. Furthermore, maternal HIV infection might also affect fetal growth [51]. The prevalence of maternal HIV infection was low in our population and did not significantly alter BW (data not shown). In areas with higher prevalence, HIV might modify the effect of malaria on fetal growth, and this should be considered in future studies.

We investigated malaria induced changes in fetal growth using both a reference population and each individual fetus as his/her own control. This approach enabled us to capture changes that may not cause LBW, but do hinder the newborn in obtaining its inherent growth potential. By utilizing ultrasound to estimate GA and a local standard weight chart for reference we circumvented some of the difficulties encountered in previous malaria studies on assessment of intrauterine growth [10], [16]. Previously, alterations in fetal growth have been evaluated using neonatal anthropometry [5], [51] or small-for-gestational-age [3], [52] as proxy measures of intrauterine growth retardation. Few previous studies have utilized ultrasound to evaluate the effect of malaria on fetal growth. One study in Congo [49], [53] showed an association between PAM and in-utero small-for-gestational-age, and a study from the Thai-Burmese border reported a decrease in fetal biparietal diameter after malaria infection [46].

To validate our ultrasound data we investigated the correlation between fetal weight gain in the different growth intervals and BW, independent of malaria infection, and found a significant correlation (data not shown). Alterations in fetal growth has been evaluated using changes in z-scores [25], [26], weight gain in grams [27], [28], or fetal abdominal circumference association with later intrauterine growth retardation [41], [54], but consensus on the best method is still to be reached. We therefore decided to combine relative weight gain and changes in z-score to evaluate the effect of PAM in our population.

One limitation of the study was that the ultrasonographs were not blinded to the women’s current or previous malaria status. Furthermore, we did not observe consistent statistically significant changes in head and abdominal circumference. This might be due the possibly decrease in accuracy when estimating fetal size using single biometric parameters [55]–[57].

In conclusion, we provide evidence that alteration of fetal growth after *P. falciparum* malaria exposure can be observed in the 3^rd^ trimester. The effects on fetal growth often occurred after a considerable delay in women who were diagnosed with malaria at the first antenatal visit and who subsequently strictly followed the antenatal program and the recommended measures to prevent malaria infections. The study was carried out in an area where malaria transmission has declined considerably and the results highlight the importance of developing interventions which can protect women early in pregnancy.

## Supporting Information

Table S1 **Comparison of included and excluded mother-newborn pairs.**
(DOCX)

Table S2 **Comparison of characteristics for malaria positive and malaria negative primi- and secundigravid mothers and their fetuses/newborns.**
(DOCX)

Table S3 **Comparison of characteristics for malaria positive and malaria negative multigravid mothers and their fetuses/newborns.**
(DOCX)

Table S4 **Characteristics of all malaria positive women.**
(PDF)

Table S5 Factors associated with 3^rd^ trimester relative fetal weight gain ((g/week)*kg) dichotomized as belonging to the lowest 25% or the highest 75% for primi- and secundigravidae.
(DOCX)

Table S6 **Factors associated Z-score at delivery among primi- and secundigravidae**. Z-score was adjusted for sex of newborn and gestational age at delivery.
(DOCX)
