# Supplementary material for: Malaria and Fetal Growth Alterations in the 3rd Trimester of Pregnancy: A Longitudinal Ultrasound Study
Source: PLoS One. 2013 Jan 11;8(1):e53794. doi: 10.1371/journal.pone.0053794 (PMC3543265; doi:10.1371/journal.pone.0053794)
Supplement: Table S1 — Comparison of included and excluded mother-newborn pairs. (DOCX) [file pone.0053794.s001.docx]

**Supplementary Table S1.** Comparison of included and excluded mother-newborn pairs.

|  |  | Included | | Excluded | |  |
| --- | --- | --- | --- | --- | --- | --- |
|  |  | Total | Median (range) / n (%) | Total | Median (range) / n (%) | *P^a^* |
| Age (y) |  | 875 | 26 (14-47) | 118 | 26.5 (16-42) | 0.86 |
| Gravidity | 1 | 876 | 175 (20.0) | 119 | 34 (28.6) | **0.004** |
|  | 2 |  | 223 (25.5) |  | 39 (32.7) |  |
|  | 3+ |  | 478 (54.6) |  | 46 (38.7) |  |
| Education ≤ primary level |  | 870 | 756 (86.7) | 119 | 90 (76) | **0.001** |
| Ethnicity | Sambaa | 87 5 | 427 | 119 | 46 | 0.31 |
|  | Zigua |  | 163 |  | 28 |  |
|  | Pare |  | 52 |  | 7 |  |
|  | Bondei |  | 35 |  | 5 |  |
|  | Other^b^ |  | 198 |  | 33 |  |
| Mat. height (cm) |  | 871 | 158 (142-186.5) | 117 | 158 (142-178) | 0.70 |
| Mat. weight at incl.(kg) |  | 870 | 53 (36.5 – 125.5) | 119 | 55 (38-102) | 0.06 |
| MUAC at incl.<23cm |  | 874 | 80 (9.2) | 119 | 7 (5.9) | 0.24 |
| HIV infection | Negative | 876 | 766 (87.4) | 119 | 78 (65.6) | **<0.001** |
|  | Positive |  | 41 (4.7) |  | 5 (4.2) |  |
|  | Unknown |  | 69 (7.9) |  | 36 (30.3) |  |

The 876 included mother-newborn pairs eligible for analysis compared with the 119 excluded mother-newborn pairs.

a) All proportions and medians were compared using Chi^2^ and Mann-Whitney test, respectively. b) Other include various ethnic groups representing <2% of the women (not stratified by gravidity).

Abbreviations: BMI = body mass index, HIV = human immunodeficiency virus, Incl. = inclusion, Mat. = maternal, MUAC = mid upper arm circumference, N = number, Y = year.
