# Supplementary material for: Malaria and Fetal Growth Alterations in the 3rd Trimester of Pregnancy: A Longitudinal Ultrasound Study
Source: PLoS One. 2013 Jan 11;8(1):e53794. doi: 10.1371/journal.pone.0053794 (PMC3543265; doi:10.1371/journal.pone.0053794)
Supplement: Table S2 — Comparison of characteristics for malaria positive and malaria negative primi- and secundigravid mothers and their fetuses/newborns. (DOCX) [file pone.0053794.s002.docx]

**Supplementary Table S2.** Comparison of characteristics for malaria positive and malaria negative primi- and secundigravid mothers and their fetuses/newborns.

|  |  | Malaria positive (n=48) | | Malaria negative (n=350) | |  |
| --- | --- | --- | --- | --- | --- | --- |
|  |  | Total | Median (range) | Total | Median (range) | *P^a^* |
| GA inclusion |  | 48 | 126 (48-158) | 350 | 129 (42-168) | 0.73 |
| GA inclusion <14weeks |  | 48 | 5 (10.4) | 350 | 71 (20.3) | 0.10 |
| GA inclusion 14-24 weeks |  | 48 | 43 (89.6) | 350 | 279 (79.7) | 0.10 |
| GA at ANV^b^ | ANV2 | 30 | 183 (174-191) | 336 | 183 (172-198) | 0.89 |
|  | ANV3 | 38 | 211 (206-224) | 330 | 211 (203-223) | 0.78 |
|  | ANV4 | 39 | 253 (235-260) | 310 | 253 (241-269) | 0.33 |
| Age (y) |  | 48 | 20 (17-35) | 350 | 22 (14-37) | 0.10 |
| Education ≤ primary level |  | 47 | 39 (83.0) | 349 | 266 (76.2) | 0.30 |
| Ethnicity | Sambaa | 48 | 21 (43.8) | 350 | 162 (46.3) | 0.23 |
|  | Zigua |  | 8 (16.7) |  | 51 (14.6) |  |
|  | Pare |  | 0 (0) |  | 31 (8.9) |  |
|  | Bondei |  | 3 (6.3) |  | 15 (4.3) |  |
|  | Other^c^ |  | 16 (33.3) |  | 91 (26.0) |  |
| Maternal height (cm) |  | 47 | 158 (143-178) | 350 | 158 (144-183) | 0.62 |
| Maternal weight at incl. (kg) |  | 48 | 53 (40-76) | 347 | 53 (37-126) | 0.39 |
| MUAC <23cm at inclusion^d^ |  | 48 | 6 (12.5) | 349 | 43 (12.3) | 0.97 |
| Received IPTp≥2 times |  | 48 | 47 (98.9) | 350 | 336 (96) | 0.51 |
| Never used bednet |  | **48** | **8 (16.7)** | **350** | **24 (6.9)** | **0.04^e^** |
| Maternal HIV infection |  | 45 | 2 (4.4) | 323 | 7 (2.2) | 0.30^e^ |
| PIH |  | 48 | 0 (0) | 350 | 13 (3.7) | 0.38^e^ |
| Diabetes |  | 48 | 0 (0) | 350 | 1 (0.03) | 1^e^ |
| Severe anemia during pregn.^f^ |  | **48** | **13 (27.0)** | **350** | **54 (15.4)** | **0.04** |
| Male newborn |  | 48 | 27 (56.3) | 345 | 172 (49.9) | 0.41 |
| Placental weight (g)^g^ |  | **35** | **540±135** | **275** | **593±142** | **0.038** |
| Head circ. at delivery |  | 37 | 345 (325-363) | 297 | 350 (283-395) | 0.09 |
| Abdominal circ. at delivery |  | 37 | 335 ((298-384) | 297 | 340 (270-387) | 0.59 |

a) Unless stated otherwise, all comparison are made using Mann-Whitney ranksum test for medians and Chi^2^ test for proportions b) Only include the women with an available fetal weight at the given visit c) Other include various ethnic groups representing <2% of the women (not stratified by malaria positivity) d) MUAC<23cm was used as a marker for poor nutritional status e) Fisher’s exact test f) ha

hemoglobin<8g/dl g) Mean±SD, Students t-test.

Abbreviations: ANV= antenatal visit, Circ. = circumference, GA = gestational age, G = gram, HIV = human immunodeficiency virus, Incl. = inclusion, IPTp = intermittent preventive treatment in pregnancy, MUAC = mid upper arm circumference, N= number, PIH = pregnancy-induced hypertension, Pregn. = pregnancy, Y = year
