# Supplementary material for: Malaria and Fetal Growth Alterations in the 3rd Trimester of Pregnancy: A Longitudinal Ultrasound Study
Source: PLoS One. 2013 Jan 11;8(1):e53794. doi: 10.1371/journal.pone.0053794 (PMC3543265; doi:10.1371/journal.pone.0053794)
Supplement: Table S3 — Comparison of characteristics for malaria positive and malaria negative multigravid mothers and their fetuses/newborns. (DOCX) [file pone.0053794.s003.docx]

**Supplementary Table S3.** Comparison of characteristics for malaria positive and malaria negative multigravid mothers and their fetuses/newborns.

|  |  | Malaria positive (n=24) | | Malaria negative (n=454) | |  |
| --- | --- | --- | --- | --- | --- | --- |
|  |  | Total | Median (range) | Total | Median (range) | *P^a^* |
| GA inclusion |  | 24 | 128 (58-167) | 454 | 131 (50-168) | 0.43 |
| GA inclusion <14weeks |  | 24 | 5 (20.1) | 454 | 72 (15.9) | 0.57 |
| GA inclusion 14-24 weeks |  | 24 | 19 (79.9) | 454 | 382 (84.1) | 0.57 |
| GA at ANV^b^ | ANV2 | 8 | 187 (182-193) | 440 | 183 (172-200) | 0.40 |
|  | ANV3 | 13 | 211 (210-217) | 438 | 211 (205-230) | 0.72 |
|  | ANV4 | 13 | 253 (251-257) | 420 | 253 (244-269) | 0.58 |
| Age (y) |  | 24 | 28 (20-40) | 453 | 30 (19-47) | 0.15 |
| Education ≤ primary level |  | 24 | 23 (82.1) | 450 | 428 (94.0) | 1 |
| Ethnicity | Sambaa | 24 | 12 (50.0) | 453 | 232 (51.0) | 0.63^c^ |
|  | Zigua |  | 6 (25.0) |  | 98 (18.0) |  |
|  | Pare |  | 0 (0) |  | 21 (4.6) |  |
|  | Bondei |  | 0 (0) |  | 17 (3.7) |  |
|  | Other^d^ |  | 6 (25.0) |  | 85 (18.7) |  |
| Maternal height (cm) |  | 24 | 156 (146-168) | 452 | 157 (142-187) | 0.64 |
| Maternal weight at incl. (kg) |  | 24 | 52 (43-89) | 451 | 54 (37-100) | 0.60 |
| MUAC <23cm at inclusion^e^ |  | 24 | 1 (4.2) | 453 | 30 (6.6) | 1 |
| Received IPTp≥2 times |  | **24** | **21 (87.5)** | **454** | **442 (97.4)** | **0.034** |
| Never used bednet |  | 24 | 0 (0) | 454 | 441 (97.1) | 1 |
| Maternal HIV infection |  | 22 | 4 (18.2) | 417 | 28 (6.7) | 0.07 |
| PIH |  | 24 | 1 (4.2) | 454 | 26 (5.7) | 1 |
| Diabetes |  | 24 | 0 (0) | 454 | 1 (0.02) | 1 |
| Severe anemia during pregn.^f^ |  | 24 | 4 ((16.7) | 454 | 84 (18.5) | 1 |
| Male newborn |  | 23 | 13 (56.5) | 447 | 218 (48.8) | 0.47 |
| Placental weight (g)^g^ |  | 14 | 655±133 | 332 | 596±151 | 0.15 |
| Head circ. at delivery |  | 16 | 350 (330-380) | 385 | 350 (301-390) | 0.46 |
| Abdominal circ. at delivery |  | 16 | 349 (322-380) | 383 | 340 (290-390) | 0.11 |

a) Unless stated otherwise, all comparison are made using Mann-Whitney ranksum test for medians and Fisher exact test for proportions b) Only include the women with an available fetal weight at the given visit c) Chi^2^ test d) Other include various ethnic groups representing <2% of the women (not stratified by malaria positivity) e) MUAC<23cm was used as a marker for poor nutritional status f) hemoglobin<8g/dl g) Mean±SD, Students t-test.

Abbreviations: ANV= antenatal visit, Circ. = circumference, GA = gestational age, G = gram, HIV = human immunodeficiency virus, Incl. = inclusion, IPTp = intermittent preventive treatment in pregnancy, MUAC = mid upper arm circumference, N= number, PIH = pregnancy-induced hypertension, Pregn. = pregnancy, Y = year
