# Supplementary material for: Malaria and Fetal Growth Alterations in the 3rd Trimester of Pregnancy: A Longitudinal Ultrasound Study
Source: PLoS One. 2013 Jan 11;8(1):e53794. doi: 10.1371/journal.pone.0053794 (PMC3543265; doi:10.1371/journal.pone.0053794)
Supplement: Table S4 — Characteristics of all malaria positive women. (PDF) [file pone.0053794.s004.pdf]

Supplementary Table S4. Characteristics of all malaria positive women.

Persistent anemia was defined as a Hb<8 g/dl at two or more ANVs. TZKH0644 suffered from asthma, none of the other women had chronic conditions. Women were only considered exposed to malaria in the period following the diagnosis. In this table fetal weight gains shown in italic are the weight gains that occurred before the malaria diagnoses. During this period it is uncertain whether the women were exposed to malaria and they were therefore excluded from the analyses presented in Table 3. Empty cells indicate that data to generate weight gains were not available. Cells with “/” represent information on each infection in women who had several infections.

ID number	Fetal growth pattern	ANV2-ANV3 (g/week*kg)	ANV3-ANV4 (g/week*kg)	ANV4-Delivery (g/week*kg)	ANV3-Delivery (g/week*kg)	Z-score at delivery	GA at inclusion (days)	GA at Delivery (days)	Gravidity	Number of infections	Timing of infection	GA at infection (Days)	Slide/PCR positive	RDT positive	Parasitemia at infection (IE/ul)	Febrile infection	Treatment	IPTp before inclusion	IPTp before 1st infection	GA (days) at 1st IPTp dose	GA (days) at 2nd IPTp dose	Sex of fetus/newborn	Ever severe anemia (Hb<8g/dl)	Persistent anemia (Hb<8g/dl)	MUAC<23cm at inclusion	Pregnancy-induced hypertension	Maternal HIV infection	Stillborn	Maternal diabetes
TZKH0013		152	196				158	267	PS	1	A2	174	N	Y	0	N	Y	N	N	174	200	M	Y	Y	N	N	N	N	N
TZKH0018					169	1,48	141	267	M	1	I	141	Y	Y	0	N	Y	N	N	210	252	M	N	N	N	N	N	N	N
TZKH0045	No		142	51	117	1,29	146	299	M	1	A2	184	Y	Y	441	N	Y	N	Y	146	212	M	N	N	N	N	N	N	N
TZKH0053	L	199			89	-0,49	135	272	PS	1	I	135	Y	Y	9384	N	Y	N	N	183	216	F	Y	N	N	N	N	N	N
TZKH0054		196					134	281	M	1	I	134	Y	Y	0	N	Y	Y	Y	133	189		N	N	N	N	N	N	N
TZKH0055	No	157			103	0,36	135	282	PS	1	I	135	Y	N	143	N	Y	N	N	195	224	M	N	N	N	N	N	N	N
TZKH0074	Im			56		-1,24	101	293	PS	1	A2	182	Y	N	486	N	N	N	N	182	213	F	N	N	N	N	N	N	N
TZKH0076	P		114	1,2	67	-1,91	127	284	PS	2	I/ I-A2	127/134	Y/Y	Y	5773/13244	N/N	N/Y	N	N	210	253	M	Y	N	N	N	N	N	N
TZKH0090		138			86		152	286	M	2	A2/D	190/286	N/N	Y	0/0	N/N	Y/Y	N	Y	152	217	M	N	N	N	N	N	Y	N
TZKH0093							127	259	PS	1	D	259	Y	Y	0	N	Y	N	Y	127	189	F	N	N	N	N	N	N	N
TZKH0109	No	*166*	*112*	99	*134*	0,68	112	285	M	1	A3-A4	245	Y	Y	45760	N	Y	N	Y	182	213	F	N	N	N	N	N	N	N
TZKH0111		232	128				122	285	PS	2	I-A2/A2	149/182	N/N	Y/Y	0/0	N/N	Y/Y	N	N	211	253	F	Y	Y	N	N	N	N	N
TZKH0157	No	*164*	*105*	*82*	*114*	-0,88	86	275	M	1	D	275	Y	Y	4163	N	Y	N	Y	142	184	M	N	N	N	N	Y	N	N
TZKH0163	P	138			49	-3,27	141	266	PS	3	I/A3-A4/D	141/242/266	Y/Y/N	Y/Y/Y	1107/22468/0	N/N/N	Y/Y/Y	N	N	141	185	M	Y	N	N	N	N	N	N
TZKH0183	Im	125	116	117	140	-0,16	95	272	M	1	I	95	Y	Y	40	N	Y	N	N	A		M	Y	Y	N	N	N	N	N
TZKH0188	P	*141*	*106*	32	*92*	-1,03	110	272	PS	1	A4	257	Y	Y	2176	N	Y	N	Y	140	186	M	N	N	N	N	N	N	N
TZKH0195	Im	111	129	75	131	0,40	140	287	PS	1	I	140	Y	N	244	N	N	N	N	140	191	M	Y	Y	N	N	Y	N	N
TZKH0270	Im	116	169	48	131		126	302	PS	1	I- A2	140	Y	Y	2565	N	Y	N	N	185	213	M	N	N	N	N	N	N	N
TZKH0313		*174*	*137*				106	269	M	1	D	269	N	Y	0	N	Y	N	Y	140	210	F	N	N	N	N	N	N	N
TZKH0317	Im	*182*	*112*	9,8	*69*	-1,78	117	292	PS	1	A4	255	Y	Y	6760	N	Y	N	Y	140	210	F	N	N	N	N	N	N	N
TZKH0351	P	*184*	*158*	13	*108*	-0,47	140	278	M	1	A3-A4	244	Y	Y	1278	N	Y	N	Y	140	173	M	N	N	N	N	N	N	N
TZKH0382	No	*136*	*147*	*89*	*155*	0,82	141	280	PS	1	D	280	Y	Y	5040	Y	Y	N	Y	141	211	M	N	N	N	N	N	N	N
TZKH0388	P	*141*	130	39	110	-1,00	125	272	PS	1	A3	210	Y	Y	2005	N	Y	N	Y	139	252	M	Y	N	N	N	N	N	N

ID number	Fetal growth pattern	ANV2-ANV3 (g/week*kg)	ANV3-ANV4 (g/week*kg)	ANV4-Delivery (g/week*kg)	ANV3-Delivery (g/week*kg)	Z-score at delivery	GA at inclusion (days)	GA at Delivery (days)	Gravidity	Number of infections	Timing of infection	GA at infection (Days)	Slide/PCR positive	RDT positive	Parasitemia at infection (IE/ul)	Febrile infection	Treatment	IPTp before inclusion	IPTp before 1st infection	GA (days) at 1st IPTp dose	GA (days) at 2nd IPTp dose	Sex of fetus/newborn	Ever severe anemia (Hb<8g/dl)	Persistent anemia (Hb<8g/dl)	MUAC<23cm at inclusion	Pregnancy-induced hypertension	Maternal HIV infection	Stillborn	Maternal diabetes
TZKH0392		149	112				94	278	PS	1	A2	187	Y	Y	0	N	Y	N	Y	141	218	M	N	N	N	N	N	N	N
TZKH0404		*176*	*111*				121	272	M	1	D	272	N	Y	0	N	Y	N	Y	144	210	M	Y	N	N	N	N	N	N
TZKH0406		*152*	*126*	*118*	*154*		137	276	M	1	D	276	N	Y	0	N	Y	N	Y	137	212	F	N	N	N	N	N	Y	N
TZKH0415	No	187	124	16	75	-1,41	138	294	M	1	I	138	N	Y	0	N	Y	N	N	189	252	F	Y	N	Y	N	Y	N	N
TZKH0419		155	112				157	265	PS	1	I	157	Y	N	206	N	N	N	N	157	211	F	N	N	N	N	N	N	N
TZKH0425		153	114				121	267	PS	2	A2/A2-A3	183/211	N/N	Y/Y	0/0	N/N	Y/N	N	Y	139	211	F	Y	N	N	N		N	N
TZKH0436	L	*181*	116	-19	51	-3,43	137	287	PS	1	A3	212	Y	Y	23588	N	Y	N	Y	137	254	M	N	N	Y	N	N	N	N
TZKH0437	L	203	92	30	72	-1,31	86	284	PS	2	I/D	86/284	Y/N	Y/Y	0/0	N/N	Y/Y	N	N	140	210	M	N	N	N	N	N	N	N
TZKH0438	No	156	140	103	161	1,48	58	282	M	1	A2	182	Y	Y	817	N	Y	N	N	A		M	N	N	N	N		N	N
TZKH0444	No	*168*	*114*	*77*	*121*	-0,06	126	280	PS	1	D	280	N	Y	0	N	Y	N	Y	140	210	F	N	N	N	N	N	N	N
TZKH0461	No	*185*	119	40	97	-1,21	109	289	PS	2	A2-A3/A4	199/254	Y/Y	Y/Y	390749/247	N/N	Y/Y	N	Y	140	210	M	N	N	N	N	N	N	N
TZKH0517		184	113				106	298	PS	2	I/I- A2	106/113	Y/Y	Y/Y	1012/665	N/N	N/Y	N	N	151	211	M	N	N	Y	N	N	N	N
TZKH0524	No	*170*	126	60	120	0,28	167	280	M	1	A3	212	Y	Y	0	N	Y	N	Y	167	212	M	N	N	N	N	N	N	N
TZKH0584	Im	*110*			122	-1,23	113	270	PS	1	A3	208	Y	Y	582	N	Y	N	Y	140	210	M	N	N	N	N	N	N	N
TZKH0618	L	211	141	24	103	-0,38	114	281	M	1	I	114	Y	Y	0	N	Y	N	N	142	211	F	N	N	N	N	Y	N	N
TZKH0638	No		113	41	90	-0,69	149	278	PS	1	I	149	Y	Y	698	N	Y	N	N	224	254	M	N	N	N	N	N	N	N
TZKH0644	Im	133	90	55	87	-1,45	156	280	PS	1	I	156	Y	Y	0	N	Y	N	N	184	210	F	N	N	N	N	N	N	N
TZKH0648	No	*148*	*135*	*58*	*115*	0,86	122	279	M	1	D	279	N	Y	0	N	Y	N	Y	141	225	F	N	N	N	N	N	N	N
TZKH0658	No	152			146	0,60	141	282	PS	1	I	141	Y	Y	0	N	Y	N	N	184	211	M	Y	N	Y	N		N	N
TZKH0678	No	168	132	83	139	0,78	121	278	PS	1	I	121	Y	Y	0	N	Y	N	N	140	210	M	N	N	N	N	N	N	N
TZKH0679	L	175	109	120	136	-0,79	128	271	PS	1	I	128	Y	Y	0	N	Y	N	N	141	212	M	N	N	N	N	N	N	N
TZKH0709		159	104				114		PS	1	I	114	Y	Y	1613	N	Y	N	N	143	218	M	N	N	N	N	N	N	N
TZKH0740	L	173	102	21	69	-0,27	153	277	PS	1	I	153	Y	Y	1024	N	Y	N	N	188	222	F	N	N	N	N	N	N	N
TZKH0775	L	214			86	-0,41	124	246	PS	1	I	124	Y	Y	457	N	Y	N	N	140	213	F	Y	N	N	N	N	N	N
TZKH0800		149	123				115	276	M	1	I	115	Y	Y	0	N	Y	N	N	140	210	M	N	N	N	Y	Y	N	N
TZKH0811	Im	140	125		139	0,53	157	258	PS	1	I	157	Y	Y	12494	N	Y	N	N	189	256	M	Y	N	N	N		N	N
TZKH0825	Im	138	130	166	182	1,93	143	278	PS	1	I	143	N	Y	0	N	Y	N	N	153	212	F	N	N	N	N	N	N	N
TZKH0838	P	119	114	23	90	-1,95	139	278	PS	1	I	139	Y	Y	0	N	Y	N	N	182	210	M	N	N	N	N	N	N	N

ID number	Fetal growth pattern	ANV2-ANV3 (g/week*kg)	ANV3-ANV4 (g/week*kg)	ANV4-Delivery (g/week*kg)	ANV3-Delivery (g/week*kg)	Z-score at delivery	GA at inclusion (days)	GA at Delivery (days)	Gravidity	Number of infections	Timing of infection	GA at infection (Days)	Slide/PCR positive	RDT positive	Parasitemia at infection (IE/ul)	Febrile infection	Treatment	IPTp before inclusion	IPTp before 1st infection	GA (days) at 1st IPTp dose	GA (days) at 2nd IPTp dose	Sex of fetus/newborn	Ever severe anemia (Hb<8g/dl)	Persistent anemia (Hb<8g/dl)	MUAC<23cm at inclusion	Pregnancy-induced hypertension	Maternal HIV infection	Stillborn	Maternal diabetes
TZKH0839	No	*180*	*124*	148	*161*	0,21	126	267	PS	2	A3-A4/A4	232/251	Y/N	Y/Y	91667/0	Y/N	Y/N	N	Y	140	211	F	N	N	N	N	N	N	N
TZKH0871	L	180	112	37	88	-0,23	119	283	PS	1	I	119	Y	Y	1509	N	Y	N	N	141	215	F	Y	N	Y	N	N	N	N
TZKH0873	L	202	156	33	113	0,74	143	290	PS	1	I	143	Y	Y	2570	N	N	N	N	182	On TS	M	N	N	N	N	Y	N	N
TZKH0878	Im	*189*	*87*	43	*74*	-1,57	126	290	PS	1	A4	256	N	Y	0	N	N	N	Y	141	222	M	N	N	Y	N	N	N	N
TZKH0901		*165*	*130*				50	275	PS	1	A3-A4	247	Y	Y	462	N	Y	N	Y	183	212	F	N	N	N	N	N	N	N
TZKH0922	P	*185*	123	16	86	-1,36	101	280	PS	1	A3	210	N	Y	0	N	Y	N	Y	140	210	M	N	N	N	N	N	N	N
TZKH0985	Im	*185*	105	43	90	-0,44	112	284	PS	1	A3	211	Y	Y	101208	Y	Y	N	Y	142	253	F	N	N	N	N	N	N	N
TZKH0999	L	174	125	20	83	-0,49	137	288	PS	1	I- A2	174	Y	Y	11091	Y	Y	N	Y	137	210	F	N	N	N	N	N	N	N
TZKH1000		200	133				112	277	PS	2	I/A2	112/182	Y/N	Y/Y	433/0	N/N	Y/Y	N	N	140	210	M	N	N	N	N	N	N	N
TZKH1019	No	*132*	*119*	*76*	*122*	-1,03	140	275	M	1	D	275	N	Y	0	N	Y	N	Y	140	212	F	N	N	N	N	N	N	N
TZKH1046	P	*144*	163	32	116	0,60	61	290	M	1	A2-A3	185	N	Y	0	N	Y	N	Y	183	211	F	N	N	N	N	N	N	N
TZKH1055	Im	145	125	15	84	-1,28	119	285	PS	1	I- A2	170	Y	Y	3206	N	N	N	Y	143	211	F	Y	N	Y	N	N	N	N
TZKH1057	No	*137*	*174*	*49*	*146*	0,25	152	279	M	1	D	279	N	Y	0	N	Y	N	Y	152	210	M	N	N	N	N	N	N	N
TZKH1063	No	168	150		138	0,54	141	272	PS	1	I	141	Y	N	86	N	N	N	N	141	212	F	N	N	N	N	N	N	N
TZKH1065		*196*	*113*				144	280	M	1	D	280	Y	Y	0	N	Y	N	Y	144	213	M	Y	N	N	N	N	N	N
TZKH1074		*158*	*122*				153	278	PS	1	A4	252	Y	Y	37471	Y	Y	Y	Y	< Incl.	211	F	N	N	N	N	N	N	N
TZKH1108	No	200	130	70	127	-0,50	78	278	PS	1	I	78	N	Y	0	Y	Y	N	N	142	212	M	N	N	N	N	N	N	N
TZKH1118	No	*153*	149	54	139	-0,13	48	274	PS	1	A2-A3	193	Y	Y	2262	N	Y	N	Y	140	210	F	N	N	N	N	N	N	N
TZKH1126	Im	152	154	105	168	2,15	115	269	M	1	A2	185	Y	Y	35475	N	Y	N	Y	140	210	M	N	N	N	N	N	N	N
TZKH1150							149	282	M	1	I	149	Y	Y	0	N	Y	N	N	N	N	F	N	N	N	N		N	N
TZKH1167	No	*192*	128	95	145	0,70	78	284	M	2	A2-A3/A3	190/210	Y/Y	Y/Y	4532/4532	N/N	Y/Y	N	Y	140	210	F	N	N	N	N	N	N	N

Abbreviations: A/ANV = antenatal visits, A = allergic to SP and did not received IPTp, D = delivery, GA = gestational age, Hb = hemoglobin, HIV = human immunodeficiency virus, I = inclusion, Im = immediate, IE = infected erythrocytes, IPTp = Intermittent preventive treatment in pregnancy, <Incl. = recieved 1st dose before inclusion in the study, M = multigravidae, MUAC = mid-upper arm circumference, N = no, No = normal, PS = primi-and secundigravidae, Y = yes.
