# Supplementary material for: Malaria and Fetal Growth Alterations in the 3rd Trimester of Pregnancy: A Longitudinal Ultrasound Study
Source: PLoS One. 2013 Jan 11;8(1):e53794. doi: 10.1371/journal.pone.0053794 (PMC3543265; doi:10.1371/journal.pone.0053794)
Supplement: Table S5 — Factors associated with 3rd trimester relative fetal weight gain ((g/week)*kg) dichotomized as belonging to the lowest 25% or the highest 75% for primi- and secundigravidae. (DOCX) [file pone.0053794.s005.docx]

**Supplementary Table S5.** Factors associated with 3^rd^ trimester relative fetal weight gain ((g/week)*kg) dichotomized as belonging to the lowest 25% or the highest 75% for primi- and secundigravidae.

|  | ANV3-ANV4 | | | | | | ANV4-Delivery | | | | | | ANV3-Delivery | | | | | |
| --- | --- | --- | --- | --- | --- | --- | --- | --- | --- | --- | --- | --- | --- | --- | --- | --- | --- | --- |
|  |  | Lowest 25% | | Highest 75% | |  |  | Lowest 25% | | Highest 75% | |  |  | Lowest 25% | | Highest 75% | |  |
|  | Total | N | Median / n | N | Median / n | *P^a^* | Total | N | Median / n | N | Median / n | *P^a^* | Total | N | Median / n | N | Median / n | *P^a^* |
| GA at inclusion | 336 | 85 | 129 | 251 | 128 | 0.37 | 282 | 70 | 134 | 212 | 126 | 0.13 | 317 | 79 | 134 | 238 | 129 | 0.41 |
| Age (y) | 336 | 85 | 22 | 251 | 22 | 0.23 | 282 | 70 | 22 | 212 | 22 | 0.82 | 317 | 79 | 21 | 238 | 22 | 0.12 |
| Education ≤ primary level | 335 | 84 | 69 | 251 | 190 | 0.22 | 280 | 69 | 57 | 211 | 156 | 0.14 | 316 | 78 | 63 | 238 | 181 | 0.39 |
| Ethnicity |  |  |  |  |  |  |  |  |  |  |  |  |  |  |  |  |  |  |
| *Sambaa* | 336 | 85 | 35 | 251 | 115 | 0.41 | 282 | 70 | 25 | 212 | 105 | 0.23 | 317 | 79 | 35 | 238 | 106 | 0.99 |
| *Zigua* |  |  | 14 |  | 40 |  |  |  | 14 |  | 24 |  |  |  | 11 |  | 33 |  |
| *Pare* |  |  | 4 |  | 24 |  |  |  | 7 |  | 21 |  |  |  | 7 |  | 21 |  |
| *Bondei* |  |  | 6 |  | 11 |  |  |  | 4 |  | 10 |  |  |  | 4 |  | 9 |  |
| *Other^b^* |  |  | 26 |  | 61 |  |  |  | 20 |  | 52 |  |  |  | 22 |  | 69 |  |
| Mat. height (cm) | 333 | 82 | 158 | 251 | 158 | 0.61 | 280 | 70 | 158 | 210 | 158 | 0.86 | 315 | 78 | 158 | 237 | 158 | 0.83 |
| Weight at incl. (kg) | 334 | 85 | 50.5 | 249 | 53 | 0.23 | 279 | 70 | 53 | 209 | 53 | 0.24 | 315 | 79 | 52 | 236 | 53 | 0.94 |
| BMI at incl. <18.5 kg/m^2^ | 331 | 82 | 15 | 249 | 30 | 0.15 | 277 | 70 | 6 | 207 | 27 | 0.32 | 313 | 78 | 8 | 235 | 32 | 0.44 |
| MUAC at incl. <23cm | **335** | **85** | **19** | **250** | **22** | **0.001** | 281 | 69 | 7 | 212 | 25 | 0.71 | 316 | 79 | 8 | 237 | 29 | 0.61 |
| Mat. weight gain (g/week) |  |  |  |  |  |  |  |  |  |  |  |  |  |  |  |  |  |  |
| *Incl-ANV3* | 334 | 85 | 194 | 249 | 219 | 0.23 | 276 | 70 | 193 | 206 | 202 | 0.40 | 315 | 79 | 182 | 236 | 219 | 0.19 |
| *ANV3-A4* |  | 85 | 250 | 251 | 286 | 0.20 | 278 | 70 | 187 | 208 | 322 | 0.74 | 281 | 66 | 209 | 215 | 182 | 0.29 |
| *ANV4-Del* |  |  |  |  |  |  | 251 | 61 | 184 | 190 | 118 | 0.94 |  |  |  |  |  |  |
| Received IPTp≥2times | 336 | 85 | 20 | 251 | 42 | 0.16 | 282 | 70 | 65 | 212 | 201 | 0.54 | 317 | 79 | 14 | 238 | 45 | 0.81 |
| HIV infection |  |  |  |  |  |  |  |  |  |  |  |  |  |  |  |  |  |  |
| *Negative* | 336 | 85 | 78 | 251 | 229 | 0.67 | 282 | 70 | 63 | 212 | 193 | 0.83 | 317 | 79 | 73 | 238 | 209 | 0.23 |
| *Positive* |  |  | 1 |  | 7 |  |  |  | 3 |  | 6 |  |  |  | 0 |  | 8 |  |
| *Unknown* |  |  | 6 |  | 15 |  |  |  | 4 |  | 13 |  |  |  | 6 |  | 21 |  |
| GA ANV3 | **336** | **85** | **211** | **251** | **211** | **0.005** |  |  |  |  |  |  | **317** | **79** | **212** | **238** | **211** | **0.003** |
| GA ANV4 | 336 | 85 | 253 | 251 | 253 | 0.41 | 282 | 70 | 253 | 212 | 253 | 0.26 |  |  |  |  |  |  |
| GA Delivery |  |  |  |  |  |  | 282 | 70 | 281 | 212 | 281 | 0.47 | 317 | 79 | 281 | 238 | 279.5 | 0.10 |
| Male newborn | 331 | 84 | 42 | 247 | 121 | 0.87 | **282** | **70** | **27** | **212** | **114** | **0.027** | 317 | 79 | 36 | 238 | 123 | 0.35 |
| Placental weight (g) |  |  |  |  |  |  | **260** | **61** | **542** | **199** | **610** | **0.008** | **288** | **70** | **527** | **218** | **604** | **0.001** |
| Place of delivery |  |  |  |  |  |  |  |  |  |  |  |  |  |  |  |  |  |  |
| *Hospital* |  |  |  |  |  |  | 282 | 70 | 63 | 212 | 200 | 0.38 | 317 | 79 | 69 | 238 | 223 | 0.061 |
| *Dispensary/other* |  |  |  |  |  |  |  |  | 1 |  | 3 |  |  |  | 1 |  | 5 |  |
| *Home* |  |  |  |  |  |  |  |  | 6 |  | 9 |  |  |  | 9 |  | 10 |  |

a) All comparison are made using Mann-Whitney ranksum test for medians and Chi^2^ test for proportions. b) Other include various ethnic groups representing <2% of the women.

Abbreviations: ANV = antenatal visit, CM = centimeter, G = gram, GA = gestational age, HIV = human immunodeficiency virus, Incl. = inclusion, IPTp = intermittent preventive treatment in pregnancy, Kg = kilogram, M = meter, Mat. = maternal, MUAC = mid upper arm circumference, N = number, Y = year.
