# Supplementary material for: Malaria and Fetal Growth Alterations in the 3rd Trimester of Pregnancy: A Longitudinal Ultrasound Study
Source: PLoS One. 2013 Jan 11;8(1):e53794. doi: 10.1371/journal.pone.0053794 (PMC3543265; doi:10.1371/journal.pone.0053794)
Supplement: Table S6 — Factors associated Z-score at delivery among primi- and secundigravidae. Z-score was adjusted for sex of newborn and gestational age at delivery. (DOCX) [file pone.0053794.s006.docx]

**Supplementary Table S6.** Factors associated Z-score at delivery among primi- and secundigravidae. Z-score was adjusted for sex of newborn and gestational age at delivery.

|  |  | Total | N | Correlation/Median z-score^a^ | *P^b^* |
| --- | --- | --- | --- | --- | --- |
| GA at inclusion |  | 334 |  | 0.03 | 0.57 |
| Age (y) |  | 334 |  | 0.05 | 0.40 |
| Education ≤ primary level | Yes | **332** | **255** | **-0.16** | **0.02** |
|  | No |  | **77** | **0.15** |  |
| Ethnicity | Sambaa |  | 154 | -0.13 | 0.57^c^ |
|  | Zigua |  | 43 | -0.16 |  |
|  | Pare |  | 30 | -0.05 |  |
|  | Bondei |  | 14 | -0.37 |  |
|  | Other^d^ |  | 93 | -0.12 |  |
| Maternal height (cm) |  | **332** |  | **0.14** | **0.01** |
| Weight at inclusion (kg) |  | **331** |  | **0.18** | **0.001** |
| BMI at inclusion (<18.5 kg/m^2^) | Yes | 329 | 42 | -0.15 | 0.70 |
|  | No |  | 287 | -0.13 |  |
| MUAC at inclusion (<23cm) | Yes | 333 | 40 | -0.24 | 0.22 |
|  | No |  | 293 | -0.10 |  |
| Mat. weight gain in (g/week) | Incl-ANV3 | 325 |  | 0.09 | 0.09 |
|  | ANV3-A4 | 315 |  | 0.08 | 0.15 |
|  | ANV4-Del | 277 |  | -0.02 | 0.77 |
| Received IPTp≥2times | Yes | 334 | 323 | -0.13 | 0.65 |
|  | No |  | 11 | -0.00 |  |
| HIV infection | Negative | 334 | 296 | -0.14 | 0.77^c^ |
|  | Positive |  | 9 | 0.17 |  |
|  | Unknown |  | 29 | -0.09 |  |
| Placental weight (g) |  | **303** |  | **0.35** | **<0.001^e^** |
| Place of delivery | Hospital | 334 | 308 | -0.09 | 0.18 ^c^ |
|  | Dispensary/other |  | 6 | -0.13 |  |
|  | Home |  | 20 | -0.56 |  |

a) Median z-score are given. When Spearman Rank correlation is used correlation coefficient is given. Z-score overall was normal distributed but not when investigated for sub-groups. Hence, non-parametric analyses were used. b) Unless stated otherwise, Mann-Whitney ranksum test was used for comparison of median z-score for groups and Spearman Rank correlation for analyses of z-score and another continuous variable. c) Kruskal-Wallis test d) Other include various ethnic groups representing <2% of the women e) Pairwise correlation.

Abbreviations: CM = centimeter, G = gram, GA = gestational age, HIV = human immunodeficiency virus, IPTp = intermittent preventive treatment in pregnancy, Kg = kilogram, M = meter, Mat. = maternal, MUAC = mid upper arm circumference, N = number, Y = year.
